# Supplementary material for: Are carbon emissions trading and green financial instruments synergistic? -Comprehensive quantitative research based on content analysis
Source: PLoS One. 2024 Mar 7;19(3):e0298601. doi: 10.1371/journal.pone.0298601 (PMC10919601; doi:10.1371/journal.pone.0298601)
Supplement: S1 Data — (ZIP) [file pone.0298601.s001.zip › Data/地级市Z_F工作报告.zip!/░▓╗╒/═¡┴Ω╩╨/└┤╫╘╣1⁄2╓┌║┼í╛┬φ┐╦╩2╛▌═°í┐_2009.docx]

——2009年1月5日在市第十四届人民代表大会第三次会议上

市人民政府市长	李 明

各位代表：

现在，我代表市人民政府，向大会报告工作，请予审议，并请市政协委员和其他列席人员提出意见和建议。

一、2008年工作回顾

刚刚过去的2008年，是极不平凡的一年。全市人民在省委、省政府和市委的正确领导下，全面贯彻党的十七大、十七届三中全会和胡锦涛总书记视察安徽重要讲话精神，以科学发展观为统领，以新一轮解放思想大讨论为动力，认真落实中央、省各项方针政策，坚定信心，团结拼搏，有效化解各种突出矛盾和问题，较好地完成了市十四届人大一次会议确定的主要目标任务，在提前八年全面建成小康社会进程中迈出了坚实的步伐。

预计，全市生产总值实现328亿元，增长14%，高于全省平均水平；财政收入突破70亿元，增长39%，总量由第六位进到第四位，增速名列全省前茅，其中地方收入23亿元，增长29%；全社会固定资产投资175亿元，增长47.5%，增速全省第二，也是2005年以来首次超过全省平均水平；城镇居民人均可支配收入15173元，增长14.4%，农民人均纯收入5580元，增长21%，增速居全省第二位；社会消费品零售总额70.6亿元，增长23.6%；新增就业1.86万人，城镇登记失业率为4%；单位地区生产总值能耗下降4.5%，化学需氧量和二氧化硫排放量分别下降1.58%和6.35%，完成省控制指标；城区空气质量好于二级以上天数344天；人口出生率8.23‰，保持低生育水平。总的来看，全市经济社会发展呈现出增长较快、质量提高、民生改善、和谐稳定的良好态势。

与此同时，我们办成了北京至福州高速铁路与南京至安庆城际铁路在铜陵交汇这一事关长远的大事，建成和开工了一批投资额10亿元以上的重大项目，为加快铜陵发展创造了更有利的条件、奠定了更坚实的基础。我们实施了一批群众多年期盼的民生工程，为70岁以上老人提供生活保障等举措在全省率先，为低收入家庭建立动态价格补贴机制的经验在全国推广。我市还顺利通过了作为全国社会治安综合治理优秀城市验收考评，获得了省社会治安综合治理先进市、省首批文明城市等称号，社区卫生服务等在全省领先，第十届中国（铜陵）青铜文化博览会成功举办，铜陵的知名度日益提高。全市人民有理由为此感到骄傲和自豪。

一年来，我们主要推进了以下六个方面的工作。

（一）大力推进新型工业化，促进经济协调发展。沉着应对急剧变化的经济环境，针对年初以来物价过快上涨、企业生产成本上升等情况，强化经济运行调节，进一步完善工业强市政策体系，全年兑现扶持企业发展资金达2.3亿元，清理并削减行政事业性和经营服务性收费605项、减少征收费用6000多万元，及时协调解决电煤供应等供需矛盾。面对全球金融危机，见事早、行动快，及时组织开展“百名干部进百企”活动，帮助企业解决了一批问题；迅速落实1亿多元资金，增建担保机构2家，增加国有担保机构资本金和中小企业贷款贴息规模，全年新增各项贷款45亿元，贷款增量、增速均位居全省第三，有效地缓解了中小企业融资困难。由于措施得法、应对有力，工业经济保持了平稳较快的发展势头。全年规模以上工业企业新增57户，完成增加值187.1亿元，增长15.8%。坚持不懈地谋划和储备重大项目，通过安排专门补助等推动项目前期工作，积极申报项目、争取资金支持，全年共获得国债和补助资金3.7亿元，成效显著。

积极打造千百亿产业。支持铜陵有色控股公司有效整合铜矿等资源，金隆35万吨电解铜扩建工程竣工，精达与美国里亚公司合资的6万吨电磁线项目进展顺利，全威铜业25万吨铜线杆、浩荣电子600万张覆铜板一期、超远72万平方米印制电路板等重点骨干项目建成或开工建设，千亿元铜产业建设成效明显。铜化集团与德国布登海姆公司10万吨精制磷酸项目前期工作取得实质性进展。铜陵海螺日产1.2万吨和上峰水泥日产5000吨水泥熟料生产线相继开工建设，建材产业规模进一步扩大。国电铜陵电厂两台60万千瓦发电机组投入运营，我省首台100万千瓦机组铜陵皖能电厂六期开工兴建，跨越我市的电力过江通道500千伏线路建成，1000千伏线路动工，电力能源基地建设加快。电子产业呈现出集群化发展态势。

积极培育新的经济增长点。巩固农业基础地位，认真落实各项强农惠农政策，全年发放政策性补贴4683万元，兑现市级公益林生态补偿资金260万元。全年粮食总产量14.6万吨，增长11.3%。新培育专业村（园区、基地）15个、市级农业龙头企业22家、各类农民专业合作组织17个，新增花卉苗木设施栽培小区、标准化蔬菜基地面积1700亩，农产品结构进一步优化，产业化经营得到新的发展。积极发展金融、物流、旅游等现代服务业。上海浦发银行铜陵分支机构、皖江农业合作银行获准设立，邮政储蓄银行挂牌成立，引进交通银行取得积极进展，第一家小额贷款公司正式营业。粮食储备加工物流园区项目实施顺利，江南文化园一期、绿源农产品大市场一期、铜陵大市场二期等工程相继竣工，逸顿国际大酒店建成开业。新增批发零售、住宿餐饮和房地产业规模以上企业31家。通过赋予县区更多管理权限、加大民生共建和财税支持力度，推动县区加快发展；打破现有行政区划界限，整合开发区和循环经济园区，明确各类园区产业定位，推动园区建设，县区、开发区完成生产总值149亿元，占全市比重由上年38.7%提高到45%。

（二）开展全民大招商，推动改革大突破。把招商引资作为推动发展、优化结构的关键举措来抓，加强组织领导，组建了专门的招商机构，实行严格的招商引资目标责任制，制定了鼓励招商、兑现奖励等一系列政策措施，精心组织或参加了投资贸易洽谈会、徽商大会、厦洽会等重大招商活动，市领导带队招商、小分队驻点招商，全市上下形成了浓厚的招商氛围。全年新批外资注册企业5个，合同利用外资增幅全省第一；实际利用外资2亿美元，增长73.9%；实际利用市外资金100亿元，增长71%；引进千万元以上项目181个，其中亿元以上项目12个。同时，狠抓投资环境建设，通过完善土地征收管理办法、提高征地补偿金标准、加快安置房建设进度等综合措施，妥善解决征地拆迁难题，比较平稳地完成了近60万平方米房屋拆迁任务，接近前三年的总和，有力地促进了项目“落地”。对外贸易稳步发展，全年进出口总额完成34亿美元，增长16.4%，总量继续位居全省第二。全市首个公用型保税仓库及出口监管仓库获准设立。外事、侨务、外宣工作全面推进，与捷克乌斯季市等发展了友好关系，对外交往日益扩大。

深化重点领域和关键环节改革。围绕转变职能、理顺关系，在全省率先平稳地实施了行政管理体制改革，初步形成职能有机统一的大部门体制。深化投融资体制改革，组建了市建设投资控股（集团）有限公司和市工业投资控股（集团）有限公司，探索建立“借、用、还”良性循环机制和保值增值的管理体制。拟定了财政体制改革方案，规范税收属地征管。深化农村综合配套改革，启动了农村集体土地产权改革试点、农民宅基地土地登记发证试点工作。推进集体林权改革，开展了国家现代林业示范市建设。着力推进重点企业的改革，港务集团与上海华集投资有限公司签订了战略重组框架协议；16户部门下划所在辖区企业改革进度加快。启动文化管理体制改革，全面实施经营服务性事业单位改制转企，59家涉改单位中有40家完成改革任务。加快发展非公有制经济，坚持扶优扶强，积极推动全民创业。全年新注册民营企业966家、个体工商户4630户，分别增长22%和16%，非公有制经济占全市经济比重继续提高。创新中小企业融资服务平台，农村信用社改革取得阶段性成果，在全国率先创设了“金融超市”，累计融资9.4亿元；开发了动产质押等新的金融产品，初步建立起银企合作长效机制。

（三）着力推进城乡大建设，加快城乡统筹发展步伐。积极推进环城北路、沿新大道、滨江大道等城市外环线建设，开工兴建西湖立交桥、青霞路立交桥等关键控制性工程，实施芜铜路、铜官大道等城市主干道扩建工程，加快了构筑城市大框架的步伐。新火车站站房、站前广场动工兴建，陵江大道等总里程达33.4公里的12条道路全线展开，全面拉开了西湖新区大建设的序幕。继续改造提升主城区功能，完成了天井湖西湖驳岸整治、城东路路面改造和观湖广场玉带河三期工程，改造了铜陵长江大桥南引道和南大门环境，修建了笔架山广场人行天桥，整治了一批支次道路，天然气置换改造基本完成，城市园林景观建设取得新进展。沿江高速全线通车，南京至安庆城际铁路开工，北京至福州高速铁路、庐铜铁路等重大项目前期工作取得突破性进展，铜陵港锚地一期工程、集装箱码头一期工程投入运行，我市区域交通枢纽地位进一步巩固和提升。

积极探索统筹城乡发展新途径，研究出台了推进城乡一体化发展建设综合配套改革试验区方案，成为省城乡一体化综合配套改革试点市。东部城区顺安片区等控制性规划编制完成，起步区建设开始启动；南部城区起步区建设规划工作基本完成。市财政投入1400多万元，继续推进新农村十项工程建设。完成了全市村庄布点等规划编制工作；建成了顺牌路和34公里通乡公路，兴建了天店路、钟顺路改造工程，开工了东联圩泵站、狼尾湖泵站、水库标准化建设、农村河道清淤整治等水利工程；新增农村沼气用户3350户，解决了5.6万农村人口饮用水安全问题；村级组织活动场所建设全面完成，农村电网、通讯等设施继续得到改造，农村生产生活条件进一步改善。

（四）狠抓科技创新和节能减排，不断增强可持续发展能力。在科技创新方面，成立了全国第一个铜产业产学研合作联盟，与中科院、江西理工大学建立了全面合作关系，签约并实施了30多项产学研合作项目，国家环境光学监测仪器工程技术研究中心产业化基地等重大科技成果产业化项目落户我市；中科大铜陵科技创业园二期投入使用，国家铜铅锌产品检测中心建成，搭建了省级铜加工工程技术研究中心等新的科技平台；实施省以上科技计划57项，其中高新技术项目38项，全市高新技术产业总产值增长19.2%；推进国家知识产权试点市建设工作，其中专利申请量273件，实现成倍增长；完善自主创新激励政策，重奖了科技创新人才。在节能减排方面，全面落实淘汰落后产能引导政策，完成了亚星焦化最后一座焦炉的关闭工作，拆除中心城区38台燃煤锅炉；组织实施重点节能项目28项、重点企业污染治理项目11项，完成西湖污水处理厂主体工程。坚持把发展循环经济作为促进资源节约和环境友好的战略举措，启动了循环经济示范市规划编制工作，大力建设滨江循环经济工业试验园，有色循环经济组团120万吨铁球团、6万吨碳酸二甲脂一期等项目建成投产，纺织服装城竣工厂房6.5万平方米，中信泰富新亚星焦化一期、方圆化纤等项目建设进度加快。铜化磷石膏综合利用项目开工，横港循环经济工业示范区建设启动。农业循环经济试验区建设继续推进。城市生活垃圾焚烧处理工程动工，海螺低温余热发电、上峰余热发电、有色年处理4000吨铜阳极泥等项目建成或基本建成，资源综合利用率、环境效益得到提高。

（五）扎实推进民生工程建设，促进城乡公共服务均等化。我们坚持以人为本，把新增财力70%以上用于民生，在省里实施18项民生工程的基础上，增加12项内容，共投入3.8亿元，精心组织实施了30项民生工程，惠及66万多城乡居民。

在就业和社会保障方面，全面落实困难群体就业优惠政策，积极为困难人员和残疾人就业提供帮扶，建立了零就业家庭动态消除机制；对3900多名城镇下岗失业人员、8500多名农民工进行了就业技能培训。及时落实企业退休人员基本养老金调标政策，妥善解决了未参保集体企业退休人员基本生活保障问题，完善了失业、医疗、工伤和生育等社会保险，提高了失业人员保险金、农民合同制工人生活补助金、被征地农民养老保障金标准；城市与农村低保标准分别由246元/月和110元/月提高到261元/月和134元/月，人均救助水平保持在全省首位。完善农村五保供养制度。建立并实施了临时困难救助制度。为319名贫困白内障患者免费实施了复明手术，启动了全国残疾人示范市争创工作。投入4500多万元用于增加粮油储备和平抑物价，为低收入家庭直接发放价格补贴资金1100多万元。

在教育方面，投入近亿元，完成了计划安排的20所农村中小学校舍标准化改造，消除了2.28万平方米中小学各类危房（其中D级危房1.5万平方米），免除了全市义务教育阶段学生学杂费，扩大了高中阶段困难家庭学生的资助范围，并在全省率先为义务教育阶段教师（含离退休人员）发放了津贴。留守儿童关爱工作得到加强。学前教育、义务教育发展良好，高中阶段普、职教育学生比例保持均衡，铜陵职业技术学院新校区正式开工，成人教育、高等教育有新发展。

在卫生方面，新型农村合作医疗人均筹资标准由50元提高到100元，参加合作医疗农民受益水平进一步提高；扩大城镇居民医疗保险参保范围和医疗救助范围，实现城镇居民医保全覆盖、城乡困难群众医疗救助全覆盖；购买公共卫生服务，城区37.5万人受益，覆盖率达91.5％；年初确定的乡镇卫生院、村卫生室、社区卫生服务机构、计生服务所标准化建设任务如期完成；大通镇血吸虫病传染源控制综合治理项目全面实施；艾滋病、结核病等重大疾病防控措施有效落实。市中医院新址建设正式启动。落实计划生育奖励政策，提高了农村计生奖励对象的奖励标准。

在文化方面，建成了一批村级文化活动室、农家书屋、社区文化活动室；完成了铜文化博物馆新馆规划选址工作；人民电影院建成开业，铜都广场公益性文化设施改造工程投入使用；在农村和市区建成了一批体育健身工程。成功举办了市第十届运动会暨第二届体育大会。

在住房保障方面，开工建设经济适用房21万平方米，改造危旧住宅13万平方米；铜矿老居民区改造、露采和郊区“两矿一公司”棚户区、铜钢公司和亚星焦化集资建房项目等按计划实施。落实实物配租房源571户，发放住房租赁补贴5633户，均超额完成省政府下达的目标任务。

（六）妥善处理突发公共事件，努力促进社会和谐。面对历史罕见的持续低温雨雪冰冻灾害，全市上下众志成城，千方百计保畅通、保供应、保民生、保稳定，最大限度地减少了灾害造成的损失，铜陵长江大桥成为雪灾期间我省连通大江南北的唯一通道。迅速有效地开展了手足口病阻击战和“问题奶粉”处置工作，把疫情等影响控制在最低程度。汶川特大地震发生后，我们视灾情为命令，全力以赴开展救援行动，立即派遣医疗、防疫小分队奔赴灾区，动员组织募集款物累计3437万元，积极落实对口援建任务，为支援灾区人民抗震救灾作出了应有的贡献。

大力推进社会治安防控体系建设，确保奥运顺利、百姓平安。全面加强公安“三基”工作，适时开展了一系列专项整治、打击行动，全市刑事案件发生率继续下降，人民群众的安全感进一步增强。加强“五五”普法工作，实施了法律援助工程。着力构建大信访格局，深入开展重信重访专项治理和市、县区领导逐级下行联合大接访活动，推动信访形势明显好转，全年群众信访件(次)下降31%。扎实开展“解决问题月”活动，集中解决了一批群众反映的实际问题。高度重视安全生产工作，完善并严格落实各级安全生产责任制，扎实开展了隐患排查治理、重点领域安全专项整治等工作，安全生产形势保持稳定。

深入开展文明创建活动。继续推进社会主义核心价值体系建设，积极开展文明行业、单位、村镇和社区创建活动。整体完成小区移交，完成21个社区亮化和一批小区整治工作，提高了社区专职工作者生活待遇并建立了自然增长机制，增加了一线环卫工人工资，“四优”和谐社区建设取得新的进展。投入2000多万元，整合客运资源，增加公交线路，更新和添购公交车辆。投资近1000万元，改善了环卫设施条件。完成广播电视村村通工程年度目标，新建广电转播台投入使用。开展了第二次全国经济普查工作。新编《铜陵市志》即将出版。文学艺术、哲学社会科学进一步发展，防震减灾、地质、气象、双拥、国防后备力量和人民防空建设等工作得到加强，民族、宗教、档案以及妇女、儿童、老龄等社会事业取得新成绩。

各位代表！过去的一年，我们自觉接受人大法律监督与工作监督、政协民主监督以及社会各界的监督，按时办结市人大代表建议203件、市政协提案258件，办理质量继续提高。我们围绕建设服务型政府，强化政务服务中心服务功能，建立了领导现场办公制度和窗口首席代表制度，试行“两集中、两到位”和基本建设项目收费“一表清”制度，进驻中心项目由上年118项增加到225项，办理各类审批（服务）事项达50多万件，行政窗口单位服务全面提速。开通了市政府信息公开网，实施了行政执法权力公开运行试点，促进了政务公开。深入开展反腐倡廉制度建设推进年活动，规范行政自由裁量权，完善政府投资管理办法和财政专项资金审批程序，建立了统一的招投标中心，强化审计监督，惩治和预防腐败体系不断完善。发挥行风热线、市民论坛等载体作用，切实解决群众反映的突出问题。全面推行“一线工作法”，进一步加大效能监察力度，政府自身建设得到加强，工作作风有了转变，服务效能进一步提升，政务环境总体上有新的改善。

各位代表！过去的一年，我们付出的努力非同寻常，取得的成绩确实来之不易。这是全市上下团结奋斗、共克时艰的结果。在此，我代表市人民政府，向全市各族人民，向广大工人、农民、知识分子和干部，向驻铜人民解放军、武警官兵和政法干警，向各民主党派、各人民团体和社会各界人士，致以崇高的敬意！向关心、支持铜陵发展的省直各部门、兄弟市和海内外友好人士，向在我市创业的境内外投资者、建设者，表示衷心的感谢！

在肯定成绩的同时，我们也清醒地认识到经济社会发展中存在的困难和问题。主要是：由于外部环境的急剧变化，我市产业结构层次较低，企业自主创新、开拓市场、抵御风险能力不强，对资源、价格过于依赖等长期积累的深层次矛盾进一步暴露，部分行业和企业发展面临较大困难，经济增长面临严峻考验；由于主导产品价格下跌和增值税转型政策的实施，我市财政收入将受到严重影响；由于停产半停产企业增加、企业生产经营效益下滑，给社会就业和城乡居民增收带来新的困难；由于历史原因，我市高能耗、高排放产业比重高，尽管采取了很多措施，但经济发展与环境容量之间的矛盾仍然比较突出，节能减排和环境保护任务十分艰巨。政府工作也还存在不少差距和不足，一些部门和少数干部主动配合、沟通协调不够，有的工作还停在嘴上、浮在面上，有的工作执行不力、效率不高，官僚主义、铺张浪费现象还不同程度地存在。对此，我们一定高度重视，采取切实有效措施加以解决。

二、2009年主要工作

2009年，是我市深入学习实践科学发展观、保持经济社会又好又快发展、提前八年全面建成小康社会的关键一年。一方面，我市面临的外部经济环境十分严峻，全球金融危机带来的不利影响还有可能加深，未来的挑战有可能更大，不确定、不稳定因素增多，我们必须切实增强危机感和紧迫感，做好应对更加复杂、更加困难局面的准备。另一方面，经过多年坚持不懈地发展，我市的发展基础更为稳固，综合经济实力和应对危机的能力明显增强，支撑我市经济发展的中长期因素没有改变，全市上下加快发展的强烈愿望没有改变。同时，国家实施积极的财政政策和适度宽松的货币政策，为我市提供了十分难得的发展机遇。只要我们坚定信心，科学应对，趋利避害，奋发进取，就一定能够克服前进中的各种困难，保持平稳较快的发展速度，并为未来发展积蓄更多的能量。

今年政府工作的总体要求是：高举中国特色社会主义伟大旗帜，全面贯彻党的十七大、十七届三中全会和胡锦涛总书记视察安徽重要讲话精神，按照省委、省政府要求和市委的部署，以深入开展学习实践科学发展观活动为动力，抢抓率先发展新机遇，善谋跨越发展新举措，增创科学发展新优势，开创和谐发展新局面，为提前八年全面建成小康社会奠定更加坚实的基础。

综合各种因素考虑，今年经济社会发展的主要目标初步安排为：地区生产总值增长11%以上；地方财政收入增幅高于地区生产总值增幅；全社会固定资产投资增长25%以上；城镇居民人均可支配收入和农民人均纯收入分别增长10%和11%以上；社会消费品零售总额增长16%以上；居民消费价格指数涨幅低于全省平均水平；新增就业1.8万人，城镇登记失业率控制在4.5%以内；单位地区生产总值综合能耗下降4%，化学需氧量和二氧化硫排放量完成省控制目标；人口自然增长率控制在6.5‰以内。为此，我们将重点做好以下九个方面工作：

（一）千方百计抓好项目投资和招商引资工作。坚持实施项目带动战略，加大有效投入力度。统筹安排投资亿元以上的重点项目50个，力争带动全社会固定资产投资240亿元以上。根据国家产业政策导向，全力以赴编报一批新项目，争取国家投资。着眼长远，加强项目谋划工作，组建若干个项目谋划小组，提出一批牵动力强、科技含量高、市场前景好、事关长远的重大项目，力争“861”计划项目储备规模达到1000亿元以上。强化对项目投资工作的领导，建立分工负责、上下联动、部门协作、务实高效的工作机制，着力推动项目前期、开工建设、竣工投产等各个环节的工作，确保项目建设顺利推进。

进一步加大招商引资力度。强化招商引资目标责任和激励机制，做到市级领导、各部门、各重点企业都有目标任务。不断创新招商方式方法，实行县处级领导带队驻点招商，组织招商专员全脱产招商，聘请招商顾问重点招商。力争在铜产业、先进制造业、高新技术产业、现代服务业和农业、社会事业等重大项目引进上有新的突破。争取引进2—3家在国内外有影响、投资额在10亿元以上的铜深加工项目，3—5家总投资5亿元以上的三产类项目。继续加强市经济技术开发区等园区基础设施建设，进一步优化招商环境，减少环节，简化程序，加强协调，搞好服务，确保引得来、落得下、发展好。力争全年实际利用内资180亿元、外资2.6亿美元，分别增长80%和30%以上。与此同时，抓好外贸基地与海关监管场所建设，落实鼓励出口政策，支持企业“走出去”开发矿产资源和开展投资合作。继续做好外事、侨务和外宣等工作，组织开展好日全食天文观测等活动，进一步提高我市知名度。

（二）坚持走新型工业化道路保增长增后劲。牢固树立保增长首先要保企业的意识，千方百计保持工业经济平稳较快发展。加强分类指导，实行一企一策，用足用活政策，确保扶持企业发展的各项政策落到实处。综合运用财政、金融、价格等工具，继续发挥“百名干部进百企”作用，切实解决企业市场销售困难、融资瓶颈加剧、成本压力过大等实际问题，积极引导企业生产适销对路产品，支持企业积极拓展销售渠道，开拓国内市场和扩大出口。进一步清理削减行政事业性收费，增加收费透明度，切实减轻中小企业负担。鼓励支持有条件的企业做好上市工作，加大利用资本市场力度，支持企业发行债券、短期融资券和中期票据，完善“金融超市”等银政、银企合作平台，力争全年各项贷款增加60亿元以上。强化经济运行监测，进一步加强煤电油运等生产要素的供需衔接，保障企业正常生产经营需要。加强创业指导，有针对性地为企业提供信息、科技等服务，促进企业增加科技含量，增强自主创新能力。

继续打造“千百亿”工程。开工建设华纳电子铜箔、锐展变速箱、超彩钛白粉扩建、铜化精制磷酸等一批重点工程，建成投产精达铜漆包线、超远印制电路板、铜陵车辆厂车轴扩建等一批重大项目，加快铜陵海螺日产万吨熟料生产线、铜陵皖能电厂六期、中信泰富新亚星焦化二期工程等一批项目建设进度，积极推进国电铜陵电厂二期、铜冠机械重型矿冶装备、三佳公司涡轮离心机、特种钢材、新型智能阀门、染料敏化太阳能电池等一批项目前期工作。鼓励有色、铜化、精达以及中小企业以增值税转型改革为契机，在规避风险的同时，开展资源整合和资产重组，加快技术改造升级，增加总量，增强竞争力。

（三）积极推动服务业加快发展。大力发展生产性服务业。加快发展现代金融保险业，力争再引进1—2家银行机构或股份制商业银行，新增担保公司3-5家；探索组建村镇银行，完善农村小企业贷款担保体系，加强农业政策性保险试点工作。大力发展现代物流业，推进横港物流园区、新火车站片区物流园建设，重点支持3—5家专业物流基地，鼓励发展第三方物流。继续抓好“万村千乡”市场工程建设，全面提升为农服务水平。积极培育信息、设计、创意、会展等新型业态，带动服务业整体水平提高。

全面提升生活性服务业。完善城市服务功能，加快推进老城区中心商务区及步行街等一批特色商业街区建设。落实促进住房合理消费的政策，推动房地产业稳步健康发展。改善住房供应结构，加大保障性住房建设力度，提供廉租住房400套；加快老铜矿、露采棚户区改造，启动扫把沟区域、园林社区和立新煤矿等棚户区和老旧小区改造；加快经济适用房建设，切实解决城市中低收入家庭住房困难。积极培育旅游市场主体，加快推进国家矿山公园、大铜官山主题公园等现代大型旅游项目建设，着力打造铜文化、工业旅游等精品线路。落实“家电下乡”等鼓励消费的政策措施，加强农村售后服务体系建设，扩大农村消费需求。继续培育文化、休闲、健身等消费热点，增强消费对经济增长的支撑作用。

（四）继续推进城乡大建设大发展。强化以铁路为重点的交通枢纽建设。积极做好南京至安庆城际铁路建设服务工作，努力推进北京至福州高速铁路、庐铜铁路前期工作并争取早日开工建设。实施畅通工程，建成西湖立交桥、青霞路立交桥和沿江高速连接线，逐步打通“断头”路，消减“丁字”路，建设市区人行立体通道，开工建设铜都大道三期工程等，改造一批城市支次道路。加快西湖新区和东、南部城区起步区建设。建成新火车站站房及站前广场、太阳历广场，整治乌木山山体，打通沿新大道、环城北路西湖新区段和滨江大道北段等骨干路网，全面拉开西湖新区路网框架，同时整治西湖环境、启动西湖公园建设。开工建设东部城区起步区骨干路网和政务文化中心；启动南部城区起步区建设。继续推进环天井湖地区和滨江地区生活岸线整治，进一步美化城市环境。

推动公共基础设施向农村延伸。积极推进城市供水、供气、供电、道路、通讯等设施与东、南部城区一体建设。加快天店路、钟顺路建设步伐。完成100公里河道清淤任务。建成西联圩、东联圩泵站。再解决3.41万农村人口饮水安全问题。建设农村户用沼气等国债项目。实施一批中心村规划建设示范工程。完善为民服务全程代理机制，全年培训农村劳动力2万人。开展“净化家园、美化环境”农村环境大整治行动，推动教育、文化、卫生、体育等公共服务事业向农村覆盖，让农民共享社会发展成果。

推动县区经济加快发展。坚持以城带乡、以工促农，积极引导城市工商资本向农村流动，鼓励城市专业人才到农村创业。积极发展农副产品加工业和配套产业，着力培育一批特色园区。促进现代农业增产增效。培育“一村一品”市级示范村10个以上，新建改造标准化蔬菜基地1000亩以上，新增白姜种植面积5000亩以上，新培育一批农业龙头企业和一批农民专业合作组织。继续抓好粮油高产攻关、农机富民、农产品标准化生产等工作，进一步增强农业综合生产能力。认真落实各项强农惠农政策，从今年起，取消涉农行政性收费，帮助农民减支增收。

（五）着力深化改革促进创新创业。进一步巩固和扩大市级机构改革成果，适时启动县、区党政机构改革。实施财政体制改革，规范财政收入划分，合理界定事权与财权。调整优化支出结构，严格控制行政性开支，实现机关公用经费“零增长”，建设节约型机关。发挥财政资金引导作用，提高资本运营效益。稳步实施增值税转型及资源税、燃油税改革，稳妥推进水、电、气等价格改革与垃圾、污水处理及排污收费改革。全面完成经营性事业单位转企改制。

全面推进城乡一体化发展综合配套改革。深化土地承包经营权流转体制改革，积极搭建土地流转市场平台，农村承包土地流转率达到30%以上。在东部和南部城区率先建立农村房屋产权流转和宅基地置换新机制。实行城乡一元化户口登记制度，开展农村社区建设试点。继续推进集体林权制度改革，实施好全国现代林业建设示范市项目。

加强自主创新能力建设。充分发挥企业在自主创新中的主体作用，新增国家和省级以上研发机构3家以上，新增市级研发机构10家以上。增强中科大创业园孵化功能。推动多形式的产学研合作，组织实施一批重大共性关键技术攻关项目。继续推进知识产权试点市工作，加大专利成果产业化力度。实施人才强市战略，启动人才安居工程，努力培育和引进一批适用人才和高端人才。

大力促进全民创业。完善和落实发展非公有制经济的政策措施。加强领导，改进服务，促进民间资本投向政府主导和鼓励的民生工程、基础设施等建设，为非公有制经济加快发展拓展空间。积极畅通货币信贷政策传递渠道，加大信贷投放力度，为非公有制经济发展创造宽松的金融环境。安排1000万元引导资金，采取“以奖代补”形式，支持县区建设一批标准化厂房和经营场所，为再就业创业和返乡农民创业提供平台。力争新增个体工商户4000户、民营企业1500家以上，其中规模以上民营企业50家。

（六）积极促进循环经济发展和节能减排工作。加快发展循环经济。全面推进滨江循环经济工业试验园基础设施和入园项目建设。推动农业循环经济试验区加快发展，加大横港循环经济工业示范区投入力度。构筑循环经济产业体系，加快铜冶炼炉渣综合利用、磷石膏综合利用、有色余热发电等工程建设，力争再开工20个以上循环经济项目。完成符合国家标准的循环经济示范市发展规划编制和审批工作。

深入推进节能减排。全面落实节能减排目标责任制。加快企业节能技术改造，实施污染减排及生态环境建设“双十工程”，开工建设危险废物集中处理中心、滨江循环园污水处理厂等项目，建成铜陵皖能电厂3#机组脱硫、城市生活垃圾焚烧处理等工程，继续关闭和淘汰小化工、小煤矿、小球团等落后生产能力；实施钟顺河、红星河及其支流水土流失与生态修复工程，开展狼尾湖和扫把沟环境综合整治，推进生态市细胞工程和环境惠民工程建设；组织修编新一轮土地利用总体规划，加强土地复垦整理，盘活存量建设用地，做到节约集约用地，促进清洁发展和可持续发展。

（七）突出抓好以民生为重点的社会建设。优先实施民生工程。按照保障类项目扩面提标、工程类项目惠及多数的原则，在省里实施28项民生工程基础上，增加17项（其中新增10项），实施45项民生工程，让全市人民共享发展成果、得到更多实惠。

完善就业和社会保障体系。实施就业“1+2”工程，对1万名下岗失业人员、返乡农民工和退伍转业军人进行就业培训，新增2万个就业岗位。进一步完善零就业家庭、零转移就业农户和困难群众的就业援助、帮扶政策。扩大城镇职工基本养老等社会保险覆盖面，开展新型农村养老保险试点。由政府“埋单”，将新型农村合作医疗筹资标准再提高30元。实施“敬老工程”，完善城区居家养老服务。推动残疾人、红十字和福利慈善事业进一步发展。

推进均衡教育向广度和深度拓展。继续实施中小学布局调整，加快校舍标准化建设，按防震要求对全市中小学校舍全面实施安全改造，新建和改造4.5万平方米。切实关爱农村留守儿童，设立引导资金，鼓励民办幼儿园改善办学条件。大力发展职业教育，加快铜陵职业技术学院新校区建设。推动高等教育、成人教育和特殊教育健康发展。加强师德教育，提升素质教育。

推进卫生事业全面发展。积极稳妥地深化卫生体制改革，推进城乡医疗卫生服务均等化。继续实施乡镇卫生院、行政村和社区卫生室标准化、农村计划生育服务机构标准化建设。完善重大疾病防控体系，提高应对突发公共卫生事件能力。实施胥坝乡血吸虫病传染源控制综合治理工程。开工建设市人民医院新门诊大楼，加快市中医院新址建设。加强和规范人口与计划生育管理工作，全面提高人口素质。深入开展爱国卫生运动，积极争创国家卫生城市。

大力加强文化建设。继续推进农村乡镇综合文化站、村级文化活动室和农家书屋建设。办好市第三届文化艺术节。加强文物、非物质文化遗产普查和保护工作，开工建设铜文化博物馆，启动图书馆新馆前期工作。新建市青少年活动中心。深化文化体制改革，大力发展文化产业。加快城乡公共体育设施建设，规划建设市体育馆新馆，办好市首届全民健身运动会。做好地方志、档案等工作。认真开展全国第二次经济普查工作。扎实推进社会主义核心价值体系建设，繁荣发展文学艺术和哲学社会科学，深入开展精神文明创建活动，积极争创全国文明城市。

（八）坚持不懈地开展平安铜陵建设。深入开展平安创建活动。强化社会治安综合治理，进一步增强人民群众的安全感。加强和改进信访工作，严格落实信访工作责任制，继续开展领导干部下行接访和带案下访活动，建立和完善有效排查和化解各类矛盾良性机制。继续推进“五五”普法活动，扩大法律援助覆盖面，做好预防未成年人犯罪工作，开展“民主法治村”、“民主法治社区”等创建活动，深入推进法治铜陵建设。

坚持安全发展。认真落实安全生产政府监管责任和企业主体责任，健全重大安全隐患、重大危险源监控制度，加大安全生产投入，强化安全隐患排查治理，坚决遏制重特大事故的发生。完善应急管理体系和工作机制，提高保障公共安全和处置突发事件能力。坚持公交优先，不断加大投入，切实解决公共交通发展与需要不相适应的矛盾。开工兴建市地震信息中心和国家气象一级站项目，完成70%村级公共气象信息服务站建设。实施食品安全工程，规范市场秩序，健全长效机制，让人民群众吃得放心、用得安心。

推动和谐共进。进一步开展“四优”和谐社区建设。加大社区整治力度，全面完成社区亮化工程。支持工会、共青团、妇联、科协等人民团体参与社会管理和公共服务，切实保障妇女和未成年人合法权益。加强对台和民族宗教工作。积极支持驻铜部队建设，抓好国防动员和民兵预备役工作，推进民防疏散基地建设，提升双拥工作水平，巩固军政军民团结，进一步增强全社会的凝聚力。

（九）切实提高行政服务能力。深入组织开展学习实践科学发展观活动，牢固树立科学发展理念。当前，改革、发展、稳定都面临巨大的挑战和压力，越是在困难的时候，我们越是要坚持科学发展不动摇，越是要坚持解放思想不停步，切实转变制约科学发展的思想观念，不断完善科学民主决策机制，着力把握发展规律、创新发展理念、转变发展方式、破解发展难题，多做有利于改善民生和转变发展方式的工作，多干打基础、利长远、增后劲的事情，努力增强工作的科学性、主动性、创造性和实效性，不断提高领导科学发展的能力。

深入开展依法行政实践，自觉依照宪法和法律规定的权限和程序行使权力、履行职责。认真接受人大及其常委会的依法监督和政协的民主监督，提高人大议案、代表建议和政协提案办理质量，真诚听取各民主党派、工商联、无党派人士及各人民团体的意见，广泛接受社会公众和新闻舆论监督。落实行政处罚（决定）自由裁量权基准制度，进一步规范行政执法行为。认真贯彻落实《政府信息公开条例》，大力推行政务公开。建立健全并严格落实行政问责、过错追究等制度。加强领导干部经济责任审计，严格落实党风廉政建设责任制，推进惩治和预防腐败体系建设，真正形成以制度管人、管事、管财的长效机制。

深入推进机关效能建设，进一步优化经济社会发展环境。像抓项目一样抓服务、提效能，全面推行“两集中、两到位”，实现行政审批项目进中心，与群众生活密切相关的服务项目进中心。逐步建立乡镇、街道服务中心，形成行政服务网络体系。建立“12345”政府服务平台，加强便民服务。坚持“一线工作法”，深入基层，深入群众，切实改进领导作风和部门行风，把心思用在发展上，把精力放在落实中，把问题解决在一线。加强行政绩效评估，完善考核内容和方式，真正让企业和人民群众成为考核的主体，努力营造风清气正、人和业兴的良好氛围。

各位代表，我们面对的经济形势严峻而复杂，肩负的发展任务繁重而艰巨。让我们高举中国特色社会主义伟大旗帜，深入贯彻落实科学发展观，在省委、省政府和市委的坚强领导下，紧紧依靠全市人民，振奋精神，坚定信心，团结拼搏，埋头苦干，以铜陵率先发展、科学发展、和谐发展的新成绩向建国六十周年献礼！
